# Elimination of Leaf Angle Impacts on Plant Reflectance Spectra Using Fusion of Hyperspectral Images and 3D Point Clouds

**DOI:** 10.3390/s23010044

**Published:** 2022-12-21

**Authors:** Libo Zhang, Jian Jin, Liangju Wang, Tanzeel U. Rehman, Mark T. Gee

**Affiliations:** 1 Department of Agricultural and Biological Engineering, Purdue University, West Lafayette, IN 47907, USA; 2 Department of Agricultural Engineering, China Agricultural University, Beijing 100083, China; 3 Department of Biosystems Engineering, Auburn University, Auburn, AL 36849, USA

**Keywords:** hyperspectral image calibration, leaf geometry impacts, 3D point clouds, NDVI changing trends, SVR modelling

## Abstract

During recent years, hyperspectral imaging technologies have been widely applied in agriculture to evaluate complex plant physiological traits such as leaf moisture content, nutrient level, and disease stress. A critical component of this technique is white referencing used to remove the effect of non-uniform lighting intensity in different wavelengths on raw hyperspectral images. However, a flat white tile cannot accurately reflect the lighting intensity variance on plant leaves, since the leaf geometry (e.g., tilt angles) and its interaction with the illumination severely impact plant reflectance spectra and vegetation indices such as the normalized difference vegetation index (NDVI). In this research, the impacts of leaf angles on plant reflectance spectra were summarized, and an improved image calibration model using the fusion of leaf hyperspectral images and 3D point clouds was built. Corn and soybean leaf samples were imaged at different tilt angles and orientations using an indoor desktop hyperspectral imaging system and analyzed for differences in the NDVI values. The results showed that the leaf’s NDVI largely changed with angles. The changing trends with angles differed between the two species. Using measurements of leaf tilt angle and orientation obtained from the 3D point cloud data taken simultaneously with the hyperspectral images, a support vector regression (SVR) model was successfully developed to calibrate the NDVI values of pixels at different angles on a leaf to a same standard as if the leaf was laid flat on a horizontal surface. The R-squared values between the measured and predicted leaf angle impacts were 0.76 and 0.94 for corn and soybean, respectively. This method has a potential to be used in any general plant imaging systems to improve the phenotyping quality.

## 1. Introduction

Plant phenotyping is a comprehensive approach for assessing plant physiological traits such as leaf moisture content, nutrient level, and disease stress [1,2,3]. In recent years, hyperspectral imaging technologies have been widely applied in agriculture to implement quantitative analysis of plant traits and accelerate progress in breeding [4,5]. Due to the non-uniform intensity of lighting in different wavelengths [6], a white reference image, uniformly reflecting the illumination, is needed to calibrate the raw hyperspectral images before further processing [7]. The raw hyperspectral images can be calibrated conforming Equation (1).

(1) $$X_{\;}^{\left(cal\right)}=\frac{X_{\;}^{\left(raw\right)}-X_{\;}^{\left(dark\right)}}{X_{\;}^{\left(ref\right)}-X_{\;}^{\left(dark\right)}}$$

where, $X^{\left(cal\right)}$ is the calibrated image; $X^{\left(raw\right)}$ is the raw image; $X^{\left(ref\right)}$ is the white reference image; and $X^{\left(dark\right)}$ is the dark current image caused by the dark current in the camera sensors.

Although a flat white reference image is effective in reducing the non-uniform lighting conditions, it does not accurately reflect the lighting condition on the 3D plant canopies. It is not possible to correct for 3D structure using a flat white referencing because the white reference object cannot be positioned at the same place as all the leaves in a canopy, which could be at quite different distances from the camera. Additionally, the plants represent complex leaf geometry such as tilt angles, and the leaf geometry and its interaction with illumination severely impact the reflectance spectra of plants [8]. As discussed by Zou et al. [9], the tilt angle distribution across the leaf surface significantly influences the plant reflectance spectra and the vegetation indices such as NDVI. In addition, PROSAIL, the famous radiative transfer model in remote sensing territory, also considers the averaged leaf angle as one of the important parameters that impact plant phenotyping results [10,11].

**Figure 1:**
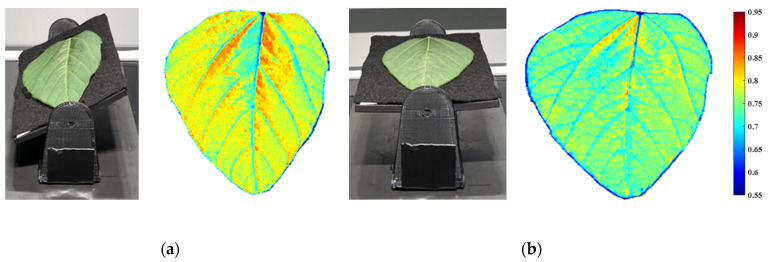
The impact of leaf angle on NDVI values. (**a**) The tilt soybean leaf and its NDVI heatmap. (**b**) The flat soybean leaf and its NDVI heatmap.

Based on the observations in our experiments, the leaf reflectance spectra and NDVI values were also impacted by leaf angles. As illustrated in Figure 1, a soybean leaf was cut off from a healthy plant (well fertilized and irrigated) and immediately scanned with two arrangements, tilt and flat, in an indoor desktop imaging system integrated with a visible-near infrared (VNIR) hyperspectral camera and an Intel RealSense depth camera. Both raw hyperspectral images were calibrated by a flat white reference acquired from a polyvinyl chloride (PVC) panel [12]. The effect of the leaf angle on NDVI values can be observed based on the NDVI heatmaps from the tilt and flat leaf. It is worth noting that the NDVI is a relevant value between the reflectance intensity in the red and *NIR* spectral wavelengths. Thus, there are no units for the NDVI values in this research.

Some plants are susceptible to the ambient environment or biochemical treatments, and their leaf angles change with time or nutrient levels. As Duncan [13], Maddonni and Otegui [14], and Gou et al. [15] described, leaf angle has a positive correlation with the interception of photosynthetically active radiation (IPAR) in corn canopies when the leaf area index (LAI) is constant. The brassinosteroids (BRs), a type of hormone in plants, play an important role in determining leaf angles; erect leaves are generated when the BRs level is low, whereas horizontal leaf angles are generated when BRs level is high [16]. According to Zhang et al. [17], the BRs contents significantly increase with N application rates in rice plants, which indicates that leaves on low-nitrogen (LN) plants drop more severely and manifest higher angles than high-nitrogen (HN) plants. As observed by Kao and Forseth [18], soybean plants perform a diurnal leaf movement, and the solar tracking attribute is influenced by different N and water availabilities. The leaf angles on water-stressed (WS) soybean plants are higher than those on well-watered (WW) plants [19]. The impacts of the ambient environment and biochemical treatments on the leaf 3D status introduce noise to the hyperspectral images and a solution is needed to remove the leaf geometry impacts.

The reflectance from a surface depends upon the direction of incident radiation, the surface radiative properties and the direction from which the surface is being viewed [20]. In the sealed hyperspectral imaging systems, the incident lighting is not fully collimated, or the reflectance on the leaf surface is not fully isotropic [21,22]. Thus, it is not precise to directly apply the bidirectional reflectance distribution function (BRDF) to simulate the impacts of leaf angles in these imaging systems [23]. As Behmann et al. [8] suggested, suitable reflectance models based on fusion of hyperspectral and 3D shape information of plants can be used to eliminate leaf geometry impacts and improve plant phenotyping quality. Further, the 3D white reference instead of the flat white reference has a potential to remove effects of plants’ 3D shape on the radiometric calibration of raw hyperspectral images [24]. However, we noticed that the reflectance intensity changes with angles in different wavelengths were not uniform for plant leaves, whereas the intensity changes with angles in different wavelengths were uniform for white reference. In other words, the shapes of leaf spectra changed at different angles, especially in visible bands, while the shapes of white reference spectra kept the same at different angles. Thus, the 3D white reference cannot completely solve the issues caused by the leaf angles, and a 3D calibration approach was developed to remove leaf angle impacts.

## 2. Materials and Methods

In this research, a 3D calibration approach based on the fusion of hyperspectral images and 3D point clouds was proposed to eliminate effects of leaf geometry and improve the practical flat white referencing calibration results. Corn and soybean experimental samples were imaged at different tilt angles and orientations to analyze the NDVI changing trend with different leaf angles. The trend was then modelled and applied to calibrate the pixels on a curved leaf to a same standard as if the leaf was flat on a horizontal surface. The entire experiment was completed in a room with a temperature of approximately 23 °C and a humidity of approximately 40%. The imaging system contained a halogen lighting source of 160 watts and the light intensity was around 3000 lumens.

### 2.1. Plant Samples

The entire experiment was comprised of two parts. In experiment part one, 40 soybean plants were grown in the Horticulture Plant Growth Facility at Purdue University (40°25′16.2″ N, 86°54′53.0″ W; https://ag.purdue.edu/hla/Hort/Greenhouse/Pages/Default.aspx, accessed on 15 February 2019). The average temperature was approximately 23 °C. The photoperiod was 14 h provided by the supplemental lighting source, and the light intensity was 100 micro mol/m^2^/s PAR to the growing area. The plants were under two genotypes (NAM_IA, Harosoy), two N treatments (300 ppm, 150 ppm), and two water treatments (WW, WS). In each treatment, there were five sample replicates. The plants were allocated in a randomized block design.

In experiment part two, the soybean and corn plants were grown in the Lilly Greenhouses and Plant Growth Facility at Purdue University (40°25′19.6″ N, 86°55′7.8″ W; https://ag.purdue.edu/LillyGreenhouse/Pages/home.aspx, accessed on 21 December 2020). The greenhouse temperature was maintained between 23 and 29 °C, and a 12-h complementary photoperiod was provided by the 600-W high pressure sodium lights. The soybean plants were from two genotypes, Pioneer and Thorne. The corn plants were under two genotypes (hybrid: B73 × Mo17, inbred: B73) and two N treatments (200 ppm, 25 ppm). All plants were well irrigated and fertilized weekly.

### 2.2. Image Acquisition of Leaf Samples at Different Tilt Angles and Orientations

**Figure 2:**
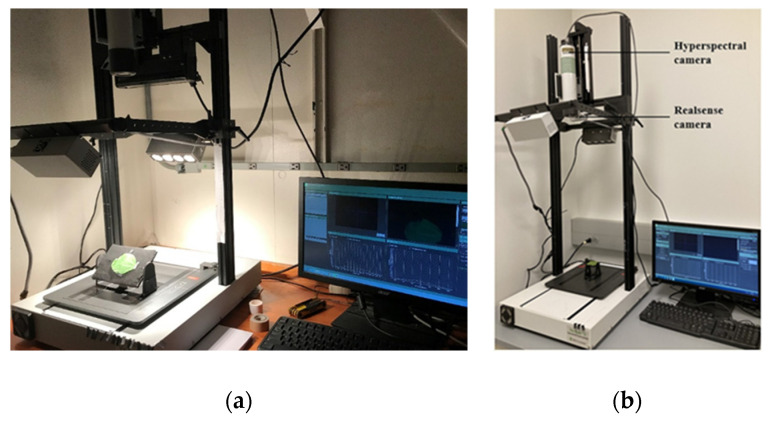
Configuration of the indoor desktop hyperspectral imaging system. (**a**) System configuration of experiment part one. (**b**) System configuration of experiment part two.

The experiment part one started when the soybean plants developed four to five trifoliate leaves. For each plant, the top-matured leaf (middle leaf of the uppermost matured trifoliate) was cut off, and immediately rotated and scanned at different tilt angles from 0° (horizontal) to 80° with intervals of 10° in the hyperspectral imaging system shown in Figure 2a. In total, 360 (40 × 9) hyperspectral images of soybean leaves were captured. The imaging system consisted of an MSV 101W VNIR hyperspectral camera (Middleton Spectral Vision Co., Middleton, WI, USA), a halogen line lighting source (eight 20-W bulbs for reflectance measurements covering 400–2500 nm wavelength range), and a Dell OptiPlex 7010 computer. The supporting, connecting, rotating, and angle-measuring parts in the hyperspectral imaging system were designed in the Solidworks 2021 software (Dassault Systèmes SOLIDWORKS Corporation, Waltham, MA, USA) and printed using a MakerBot Replicator+ 3D printer (MakerBot Industries, Brooklyn, NY, USA).

In Figure 2a, the rotation board supporting the leaf was relatively large, which might interfere the incident and reflectance lighting. The halogen lights were close to the leaf samples, which might burn and damage them during imaging. Besides the leaf tilt angles, Jay et al. [25] suggested to take the factor of leaf orientation into account in their radiative PROCOSINE model. Thus, as shown in Figure 2b, the configuration of the hyperspectral imaging system was adjusted, and a new rotation platform was designed in experiment part two.

**Figure 3:**
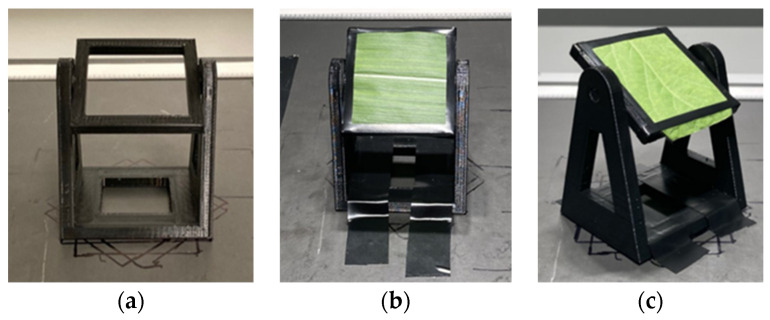
The rotation platform to capture leaf images at different tilt angles and orientations. (**a**) Structure of the rotation platform (length×width×height: 40 × 40 × 58 mm). (**b**) Corn leaf piece (40 × 40 mm) at 0° tilt orientation. (**c**) Soybean leaf piece (40 × 40 mm) at 45° tilt orientation.

To capture leaf hyperspectral images from different tilt angles and orientations, a hollow and compact rotation device was developed in Figure 3a. The leaf pieces (40 × 40 mm) pruned from the soybean and corn plants were affixed on the rotation platform and scanned at different tilt angles and orientations. For instance, a leaf piece was first settled at 0° tilt orientation in Figure 3b, rotated forward, and imaged at 9 tilt angles from 0° (horizontal) to 80° with intervals of 10°. The device was next turned to an orientation of 45° in Figure 3c, and the leaf piece was rotated forward and imaged at 9 tilt angles from 0° to 80° again. The process was repeated until one round (8 orientations from 0° to 315° at intervals of 45°) was completed. For each leaf piece, 72 (8 tilt orientations and 9 tilt angles at each orientation) hyperspectral images were collected.

The soybean plants had developed five trifoliate leaves at the time of image collection. On each soybean plant, two leaf pieces were cut, one from the top matured leaf and the other from the leaf, two trifoliate below the top matured leaf. Two soybean plants from each genotype were sampled, and thus 576 (2 × 2 × 2 × 72) hyperspectral images were collected for soybean. The corn plants were at V7 to V9 stage during image acquisition. On each corn plant, two leaf pieces were cut, one from the middle part of the top-collar leaf and the other from the leaf, two positions above the top-collar leaf. Three corn plants in each genotype and treatment were sampled, and thus 1728 (3 × 4 × 2 × 72) hyperspectral images were collected for corn.

### 2.3. Image Processing of Leaf Samples from Different Tilt Angles and Orientations

The leaf samples were segmented out from the background using a red-edge (680–732 nm) algorithm [12] in Matlab R2021a software (The MathWorks Inc., Natick, MA, USA). Heterogeneous vegetation indices can be extracted from the green leaf spectra such as NDVI, GARI (green atmospherically resistant index), CCCI (canopy chlorophyll content index), CAI (cellulose absorption index), PRI (photochemical reflectance index), and NDWI (normalized difference water index). As Wiegand et al. [26] and Bannari et al. [27] described, these vegetation indices can be used to represent the plant chlorophyll concentration/rate of photosynthesis, N status, diurnal radiation use efficiency, and crop water status. In this research, NDVI was first studied due to its wide use. For each pixel on a plant leaf, the NDVI value was calculated following Equation (2). For one leaf, all pixel NDVIs were averaged into one value to represent the chlorophyll content of the entire leaf.

(2) $$\mathrm{NDVI}=\frac{NIR-RED}{NIR+RED}$$

where, *RED* and *NIR* denote the reflectance intensity at the wavelengths of 680 and 800 nm, respectively [28].

At one tilt orientation, the NDVI values of a same leaf sample varied with the tilt angle increasing from 0° to 80°, and the ratios of NDVIs and 0° NDVI can be used to demonstrate the NDVI changing trend. In experiment part one, the soybean leaves were only rotated towards one tilt orientation (0°), whereas in experiment part two, the soybean and corn leaves were rotated towards eight orientations (0–315°). Next, the NDVI changing trends were summarized and modeled to eliminate leaf angle impacts. With tilt angles and orientations as two independent variables, a predictive modeling of the ratios was developed. Since the relationship was not necessarily linear, the support vector regression (SVR) was applied [29]. The venetian blinds cross-validation method was used to prevent overfitting [30]. If the tilt angle and orientation of a leaf pixel were known, its original NDVI could be divided by the corresponding ratio predicted from the SVR model to remove the angle impact. The process was named 3D calibration.

### 2.4. Fusion of Hyperspectral Images and 3D Point Clouds

As shown in Figure 2b, to obtain the angle information of plant leaves, an Intel RealSense depth D435 camera (Intel Corporation, Santa Clara, CA, USA) was mounted in the hyperspectral imaging system. The specific parameters of the two cameras are introduced in Table 1.

**Figure 4:**
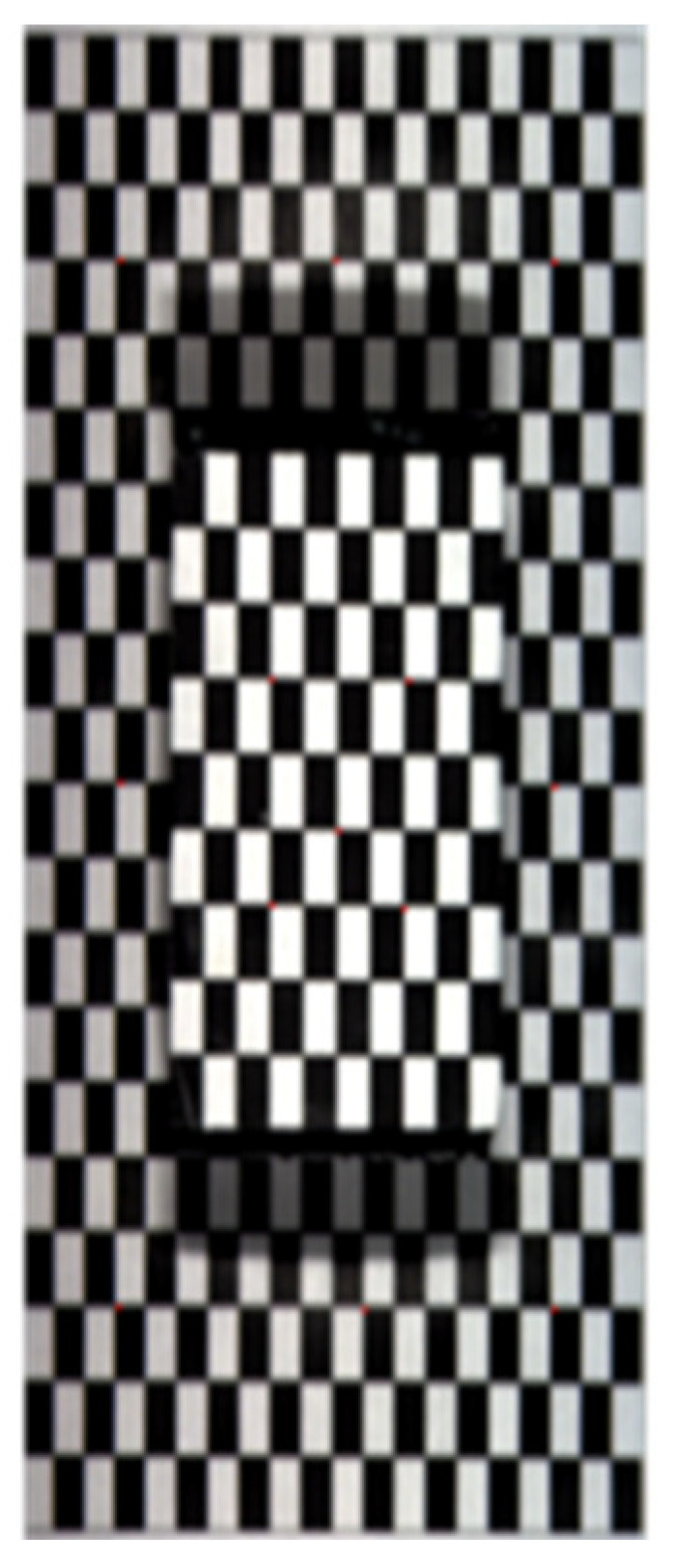
The checkerboard platform for fusion of hyperspectral images and 3D point clouds.

To fuse the hyperspectral images and 3D point clouds, a checkerboard platform (Figure 4) was imaged using both the hyperspectral camera and the depth camera. The platform contained two layers, top and bottom, with 50 mm difference in height. As Gupta and Hartley [31] described, the linear pushbroom imaging system can be interpreted as:

(3) $$\left[\begin{array}{c} u\\ wv\\ w \end{array}\right]=\left[\begin{array}{c} m_{11}\\ m_{21}\\ m_{31} \end{array}\begin{array}{c} m_{12}\\ m_{22}\\ m_{32} \end{array}\begin{array}{c} m_{13}\\ m_{23}\\ m_{33} \end{array}\begin{array}{c} m_{14}\\ m_{24}\\ m_{34} \end{array}\right]\left[\begin{array}{c} x\\ y\\ \begin{array}{c} z\\ 1 \end{array} \end{array}\right]$$

where, *u*, *v* are row and column coordinates on the hyperspectral images; *x*, *y*, *z* are corresponding ground coordinates from the 3D point clouds; *w* is a coefficient; *m_ij_* is a component in the projection matrix *M*.

The projection matrix *M* was derived in the following manner. On the hyperspectral image of the checkerboard platform, the coordinates (*u*, *v*) of the corner points were identified using a corner detection function in Matlab. The checkerboard 3D point cloud consisted of two images, the color image and the depth image. The coordinates of the corner points were extracted from the color image using the same corner detection function and then the ground coordinates (*x, y*, *z*) of the corner points were located in the depth image using the coordinates derived from the color image. Thus, for one corner point, a pair of (*u*, *v*) coordinates were integrated with a group of (*x*, *y*, *z*) coordinates. In this research, 13 corner points from both the top and bottom layers were fit in Equation (3), and the projection matrix *M* was solved conforming the direct linear transformation (DLT) and least-squares approaches. As suggested by Behmann et al. [8], a third-degree Chebyshev polynomial can be applied to diminish the effects of non-linear factors in the matrix *M* and improve the fusion quality. The resolution of the hyperspectral image was higher than the 3D point cloud. The interpolation function with the Bilinear method was used to solve the issue of resolution difference.

### 2.5. Application of 3D Calibration on Plant Leaves

To calculate the tilt angle and orientation of a leaf pixel, a small local curve was firstly simulated based on this pixel and its neighbor pixels, and then the normal vector of the simulated curve was extracted. Using the normal vector and the Equations (4) and (5) below, the pixel’s tilt angle and orientation can be obtained [32]. Combined with the predictive ratio SVR models, the 3D calibration was applied on the sampled soybean and corn leaves. The raw hyperspectral image of a leaf sample can be calibrated with three protocols: flat leaf calibrated by flat white reference (ideal calibration), curved leaf calibrated by flat white reference (practical calibration), and applying 3D calibration on a curved leaf after practical calibration. As suggested by Zhang et al. [12], the pixel-level NDVI distribution across the leaf surface was established using the kernel density estimate (KDE) and probability density function (pdf) to compare the three calibration protocols.

(4) $$\alpha =\tan^{-1}\left(\frac{v}{u}\right)\times \frac{180}{\pi }+Q$$

(5) $$\beta =\left|\tan^{-1}\left(\frac{w}{\sqrt{u^{2}+v^{2}}}\right)\times 180/\pi \right|$$

where, [*u v w*] denotes the normal vector of the simulated curve, α, β denote the tilt orientations and angles, respectively, and *Q* depends on the quadrant and is defined by *u* and *v*.

## 3. Results and Discussion

### 3.1. Soybean NDVI Changing Trend

**Figure 5:**
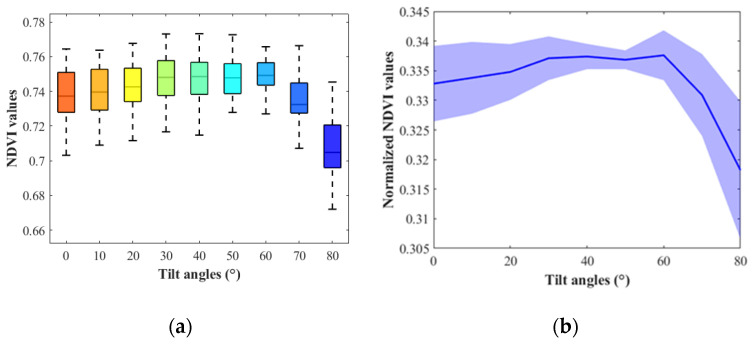
The soybean NDVI values at different tilt angles in experiment part one. (**a**) The box plots of NDVI values. (**b**) The line plots of normalized NDVI values. The ‘blue river’ denotes the 95% confidence interval.

**Figure 6:**
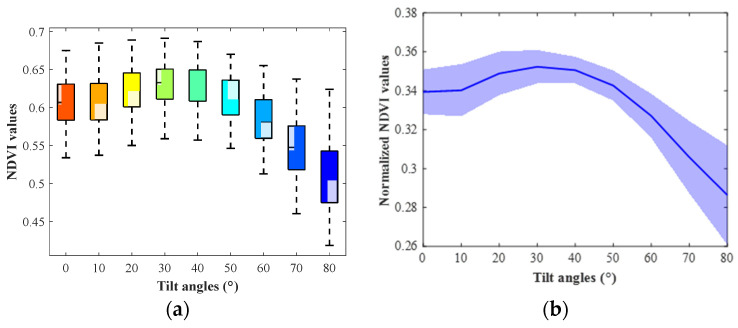
The soybean NDVI values at different tilt angles in experiment part two. (**a**) The box plots of NDVI values. (**b**) The line plots of normalized NDVI values. The ‘blue river’ denotes the 95% confidence interval.

In experiment part one, the box plots and line plots of NDVI values at different tilt angles of 40 soybean leaves were generated to demonstrate NDVI changes with angles. As shown in Figure 5, the NDVI values increased slightly and decreased largely with the angle changing from 0° (horizontal) to 80°. In experiment part two, the NDVI values of different tilt angles at all orientations were summarized. The soybean leaf NDVI increased first, culminating at approximately 30°, and then decreased dramatically with the angle rising from 0° to 80° (Figure 6). The results from both the experiment part one and two showed that soybean leaf NDVI values went up at the beginning and then declined along with the tilt angle increasing. However, the NDVI in experiment part two peaked earlier than that in experiment part one with the leaf angle from 0° to 80°, and the changing curve in experiment part two was smoother than that in experiment part one. As illustrated in Figure 3, the setting of camera and lights was adjusted, and the rotation platform and protocol were redesigned from experiment part one to part two. The major changing trends of NDVI values remained the same, increasing slightly at the beginning and decreasing rapidly at high angles, but the peaks differed between experiment part one and two. The curve from experiment part two was smoother than that from part one. Thus, the imaging system settings impacted the NDVI changing trends with angles. The leaf sample size in experiment part two was small, and more data would be collected to support our observations.

In experiment part two, the NDVI values of the soybean leaves were organized into a 64 × 9 (8 leaf samples × 8 tilt orientations × 9 tilt angles) dataset. The columns of the dataset were tilt angles from 0° to 80° at intervals of 10°, and the rows were tilt orientations from 0° to 315° at intervals of 45°. The number of replicates for each tilt orientation was 8. The two-way analysis of variance (ANOVA) was performed on the dataset. As demonstrated in Table 2, the *p*-values for the columns, rows, and the interaction between them were 0, 6.006 × 10^−5^ and 0.8846, respectively. The tilt angles and orientations significantly affected the soybean NDVI values, however, there was no evidence of an interaction effect. Both the tilt angles and orientations should be considered when analyzing the leaf geometry impacts on soybean leaves.

### 3.2. Corn NDVI Changing Trend and Comparison with PROSAIL Outputs

**Figure 7:**
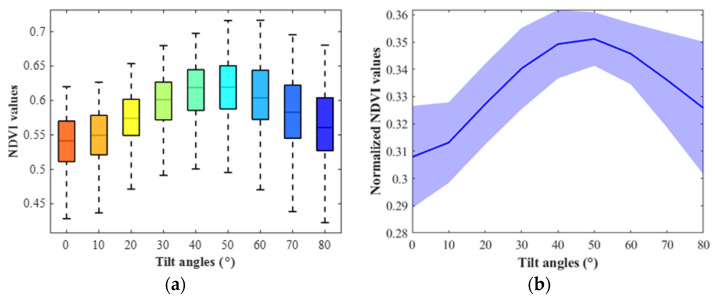
The corn NDVI values at different tilt angles in experiment part two. (**a**) The box plots of NDVI values. (**b**) The line plots of normalized NDVI values.

**Figure 8:**
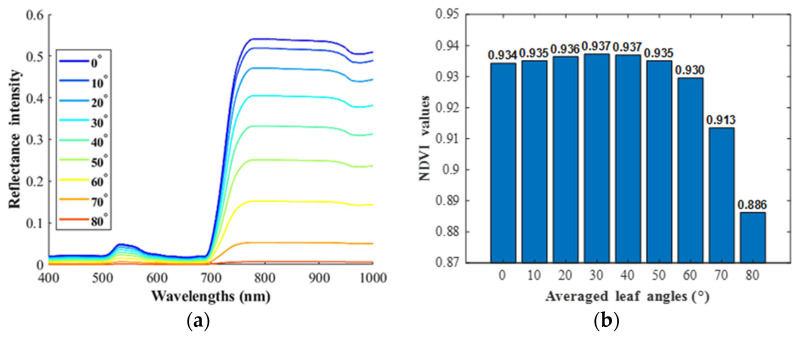
The PROSAIL model outputs for a standard corn plant. (**a**) The bi-directional reflectance spectra at different averaged leaf angles. (**b**) The corresponding NDVI values.

In experiment part two, for one corn leaf piece, the NDVI changing trend with angles from 0° to 80° was derived at each tilt orientation. As illustrated in Figure 7, the corn leaf NDVI values increased first, peaking at approximately 50°, and then decreased with the tilt angle rising from 0° to 80°. The observations were compared with outputs of PROSAIL (a radiative transfer model to estimate plant canopy biophysical variables in agriculture). The PROSAIL 5B package was used to simulate the plant reflectance spectra in the wavelengths of 400 to 1000 nm in MATLAB software. As shown in Figure 8, the reflectance spectra of a standard corn plant in PROSAIL changed at different averaged leaf angles from 0° to 80°, and the corresponding NDVI increased first and then decreased. What we observed in the indoor desktop imaging system was slightly distinct with the PROSAIL outputs, especially at the high angles. The general PROSAIL model might not fit every imaging system or cannot be applied in the indoor imaging system directly. Further, the NDVI changing trends differed between the soybean and corn species.

The NDVI values of the corn leaves were organized into a 96 × 9 (24 leaf samples × 8 tilt orientations × 9 tilt angles) dataset. The columns of the dataset were tilt angles from 0° to 80° at intervals of 10°, and the rows were tilt orientations from 0° to 315° at intervals of 45°. The number of replicates for each tilt orientation was 24. A two-way analysis of variance (ANOVA) was performed on the dataset. As demonstrated in Table 3, the *p*-values for the columns, rows, and the interaction between them were 0, 0 and 5.0877 × 10^−4^, respectively. The tilt angles, tilt orientations, and their interaction significantly affected the NDVI values. Both the tilt angles and orientations should be considered when analyzing the leaf geometry impacts on the corn leaves.

### 3.3. SVR Modelling for Soybean and Corn

**Figure 9:**
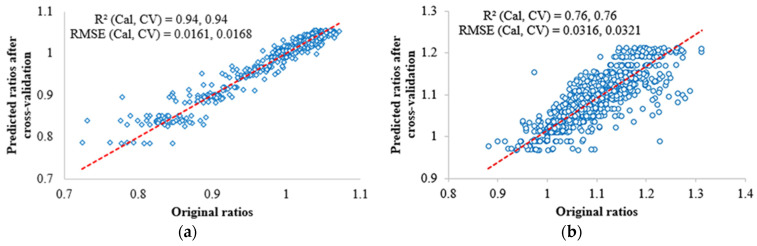
The SVR modelling results for soybean and corn in experiment part two. (**a**) Soybean model result. (**b**) Corn model result.

In experiment part two, with tilt angles and orientations as two independent variables, the SVR model was developed to predict the ratios of NDVI and 0° NDVI for both soybean and corn. The 3D calibrated NDVI can be obtained with the original NDVI from leaf pixels at different angles divided by the corresponding ratios. In this manner, the impacts of leaf angles can be eliminated. As illustrated in Figure 9, the R-squared values between the measured and predicted ratios for soybean and corn were 0.94 and 0.76, respectively. Since the leaf sample size of corn was larger than soybean, we also tried to reduce the corn data and rebuild the corn SVR model. The results still showed that the soybean performed better than the corn at SVR modelling. The higher performance in soybean may be due to flatter and more uniform leaves as compared to corn. To make the soybean and corn SVR be general models for any plants, more data needs to be collected.

### 3.4. Fusion of Hyperspectral Images and 3D Point Clouds

As shown in Figure 4, the row and column dimensions of the checkerboard hyperspectral image were approximately 1886 and 782 pixels, respectively, and the real size of the checkerboard was 200 × 200 mm. Thus, the row and column resolutions of the hyperspectral image were approximately 0.1060 and 0.2558 mm/pixel, respectively. The XYZ coordinates of the 13 corner points were extracted from the 3D point cloud of the checkerboard platform. Following Equation (3), the estimated coordinates of rows and columns of the corner points on the hyperspectral image were computed. In Table 4, the maximum row and column mismatching were 3 pixels (0.3180 mm) and 2 pixels (0.5116 mm), respectively.

**Figure 10:**
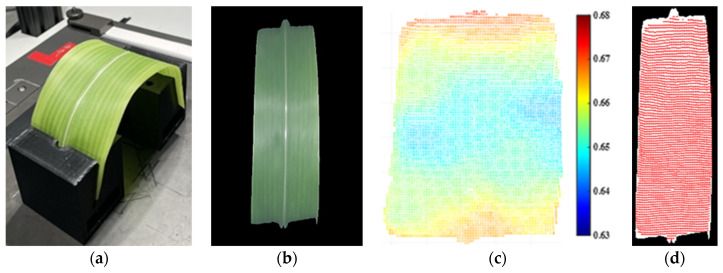
The fusion of the hyperspectral image and 3D point cloud of a corn leaf sample. (**a**) Curved corn leaf piece in the imaging system. (**b**) The segmentation of the hyperspectral image. (**c**) The segmentation of the 3D point cloud (unit: m). (**d**) The fusion of the hyperspectral image and 3D point cloud.

Due to the space limitation of the imaging system, a corn leaf piece was pruned from a healthy plant and bent slightly to an arch shape in Figure 10a. The curved leaf was imaged using both the hyperspectral camera and depth camera. Conforming Equation (3), the leaf hyperspectral image in Figure 10b and 3D point cloud in Figure 10c were fused in Figure 10d. The white pixels belonged to the hyperspectral image segmentation, whereas the red pixels were from the 3D point cloud segmentation.

### 3.5. Application of 3D Calibration on Soybean and Corn Leaves

#### 3.5.1. Soybean Leaf Calibration

**Figure 11:**
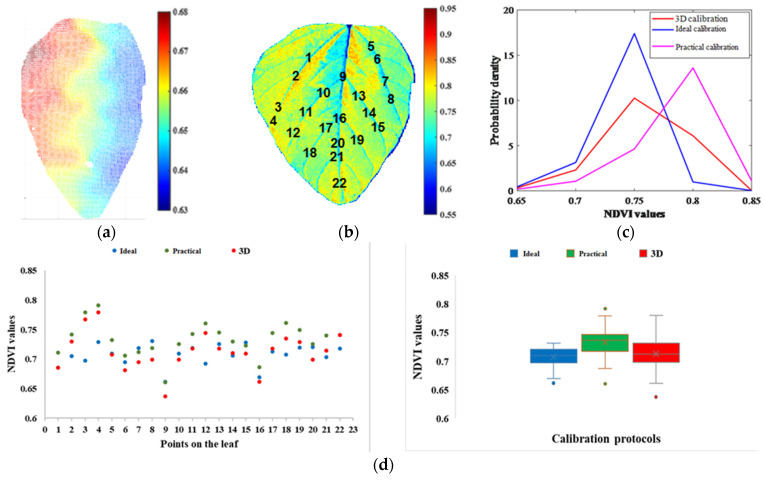
The comparisons of three calibration protocols on a soybean leaf. (**a**) The 3D point cloud of the tilt leaf (unit: m). (**b**) The NDVI heatmap after 3D calibration and 22 points manually selected using the vein structure. The points of 1–22 are marked on the figure. (**c**) The probability density curves of all the pixel NDVIs from three calibration protocols. (**d**) Scatter and box plots of 22 NDVI values manually selected from three calibration protocols.

The tilt soybean leaf in Figure 1a was also imaged using the depth camera in the adjusted indoor desktop imaging system, and the 3D calibration approach was applied on this tilt leaf. The NDVI values of the tilt leaf were larger than those of the flat leaf in Figure 1b because the NDVI increased first with angles from 0° to 80° based on the soybean NDVI changing trend. As shown in Figure 11a, the 3D point cloud of the leaf was segmented out from the background, and the right edge was closer to the camera lens (depth was around 0.63 m). The tilt angle and orientation of each pixel were calculated following Equations (4) and (5), and the corresponding ratios were predicted from the corn SVR model. In Figure 11b, the 3D calibrated NDVI heatmap was obtained with the original NDVI divided by the ratios. For this soybean leaf, there existed three calibration protocols: the flat leaf calibrated by a flat white reference (ideal calibration), the tilt leaf calibrated by a flat white reference (practical calibration), and the 3D calibration. According to the probability density curves in Figure 11c, the result after 3D calibration was closer to the ideal calibration.

In Figure 11b, by virtue of the leaf vein structure, 22 points were manually selected from the NDVI heatmaps after the ideal calibration, the practical calibration, and the 3D calibration, respectively. Centered around each point on each heatmap, 36 neighbor pixels were picked out and their NDVI values were averaged. As shown in the scatter plots and box plots in Figure 11d, the averaged NDVI of the 22 selected points from the 3D calibration heatmap was closer to that from the ideal calibration heatmap. In the box plots, the paired *t*-test *p*-values of 22 NDVI values between the ideal and practical calibration, the ideal and 3D calibration, and the 3D and practical calibration were 0.0010, 0.5164, and 0.0305, respectively. At the significant level of 0.05, there was no significant difference between the ideal and 3D calibration, whereas both of them significantly differed from the practical calibration. Thus, the 3D calibration approach proposed in this research has a potential to remove leaf angle impacts and improve plant phenotyping results.

#### 3.5.2. Corn Leaf Calibration

**Figure 12:**
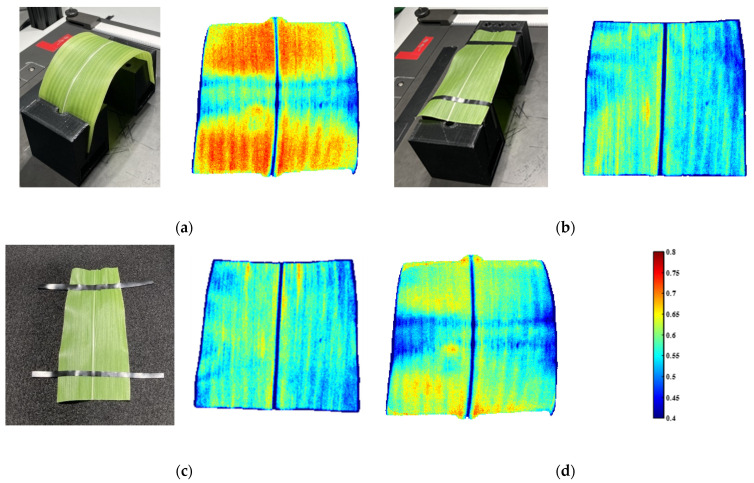
The comparisons of four calibration protocols on a corn leaf sample. (**a**) The curved leaf and its NDVI heatmap. (**b**) The suspended flat leaf and its NDVI heatmap. (**c**) The flat leaf fixed on a black cloth and its NDVI heatmap. (**d**) The NDVI heatmap after 3D calibration.

The corn leaf piece in Figure 10a was imaged with two protocols, curved and flat. The flat imaging protocol contained two arrangements with the leaf suspended in the air and affixed on a black felt fabric, respectively. As shown in Figure 12a, the NDVI values on the two sides, where the angles were high, were larger than those in the middle, where the angles were small, which was consistent with the corn NDVI changing trend above. For this corn leaf, there existed four calibration protocols: the suspended flat leaf calibrated by a flat white reference (ideal calibration 1) in Figure 12b, the flat leaf fixed on the black cloth calibrated by a flat white reference (ideal calibration 2) in Figure 12c, the curved leaf calibrated by a flat white reference (practical calibration) in Figure 12a, and the 3D calibration in Figure 12d.

**Figure 13:**
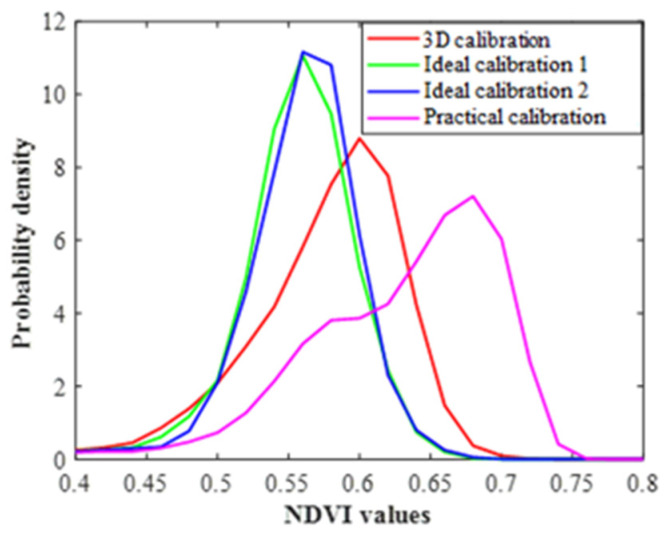
The corn leaf probability density curves of pixel NDVIs from four calibration protocols.

To demonstrate the differences of distinct calibration protocols, the probability density curve of all the pixel NDVI values across the leaf surface was generated for each of them. As shown in Figure 13, there was almost no difference between the ideal calibration 1 and 2, thus, the flat leaf imaging protocol, suspended in the air or fixed on a black cloth, did not influence the flat leaf phenotyping result significantly. The result after 3D calibration was closer to the ideal calibrations.

#### 3.5.3. Summary of 3D Calibration on Soybean and Corn Leaves

For the soybean and corn, the 3D calibrated NDVI heatmaps were derived with the proposed 3D calibration approach applied on the curved leaves to eliminate leaf angle impacts. Meanwhile, the curved leaves were stretched to be flat and horizontal to acquire the flat leaf NDVI heatmaps (standard). Firstly, the curved leaf NDVI heatmaps before and after 3D calibration were visually compared with the flat leaf NDVI. It was observed that the NDVI heatmaps changed with various leaf angles, and the changes conformed the trends from the acquisition experiment of leaf angle impact models in Figure 6 and Figure 7. Secondly, the probability density distributions across the curved and flat leaves were calculated. According to both the NDVI heatmaps and probability density distributions in Figure 1, Figure 11, Figure 12 and Figure 13, after 3D calibration, the phenotyping results were closer to the flat leaves (standard). Thus, the proposed 3D calibration has a potential to calibrate leaf pixels at different angles to a same standard and remove leaf angle impacts.

#### 3.5.4. Future Opportunities and Limitations

In this research, the proposed 3D calibration was only applied to remove leaf angle impacts on NDVI values of soybean and corn leaves. The reflectance intensity changes with angles in each spectral wavelength can also be summarized and modeled, hence the 3D calibration can be applied to remove impacts of leaf angles on the entire spectra. The evaluation of the 3D calibration was implemented using only a few soybean and corn leaf samples. A more comprehensive assessment is needed. Thus, the soybean plants subject to different genotypes and biochemical treatments will be grown, and each plant will be imaged using both the hyperspectral camera and depth camera in the indoor desktop system. The top-matured leaf (the middle leaf of uppermost matured trifoliate) will be selected in the image of each plant, and the 3D calibration can be applied on all the top-matured leaves. After done with the imaging work, the plants will be harvested to collect the ground truth data (e.g., N contents). Combined with the ground truth data, the NDVI values can be used to discriminate distinct treatments, while the spectra can be used to build statistical models of nutrient elements. The discrimination and modelling results before and after 3D calibration on the top-matured leaves will be compared to more thoroughly study the impacts of leaf angles.

The proposed 3D calibration was limited inside the indoor desktop imaging system, where the halogen lights were the only lighting source. In this paper, our model only applies to non-shaded leaf areas. For our research in the indoor desktop imaging system, the top-matured leaf was barely shaded. In other scenarios when shades exist in the image, we recommend segmenting out the shaded area before moving forward with other image processing algorithms and modeling. Because of the configuration differences, the models need to be rebuilt for various imaging systems, which affects the migration ability of the models. In addition, more soybean and corn leaf samples under different genotypes and biochemical treatments (e.g., N and water) should be collected to improve the preciseness of the models. In the field environment, we believe the leaf slope is one source of variability in crop remote sensing, among many other co-existing sources such as ambient lighting, wind speed, temperature, time of the day, and so on. We have built calibration models for the other sources of variability. Two examples can be found in the published papers [33,34]. In this paper, we are focused on only calibrating the slope factor, and hopefully by combining this with the other previous models, we will keep improving the remote sensing quality.

## 4. Conclusions

Based on the fusion of hyperspectral images and 3D point clouds, a 3D calibration approach was developed to remove leaf angles impacts on plant reflectance spectra and vegetation indices such as NDVI. The NDVI values were influenced by both the leaf tilt angles and the tilt orientations. The NDVI changing trends with angles differed between the soybean and corn species. Although both of them showed an initial increase followed by a decrease in NDVI values as the tilt angle rises from 0° (horizontal) to 80°, the soybean NDVI peaked earlier than the corn NDVI, and the soybean NDVI at high angles dropped below the 0° NDVI, whereas the corn NDVI at high angles remained above the 0° NDVI. With the tilt angles and orientations of plant leaves as two independent variables, the SVR models were built to predict the ratios of NDVI values at distinct angles and 0° NDVI, and the R-squared values between the measured and predicted ratios for soybean and corn were 0.94 and 0.76, respectively. For leaf pixels, their tilt angles and orientations can be calculated based on the 3D point clouds, and the corresponding ratios can be predicted from the SVR models. With the original NDVI values divided by the ratios, the leaf pixels at different angles can be calibrated to a same standard as if the leaf was laid flat on a horizontal surface. A few soybean and corn leaf samples were used to evaluate this proposed 3D calibration. For each leaf, there existed three calibration protocols: the flat leaf calibrated by a flat white reference (ideal calibration); the curved leaf calibrated by a flat white reference (practical calibration); and the 3D calibration. According to the probability density curves of the pixel NDVI values from the three calibration protocols, the results after 3D calibration were closer to those from the ideal calibration. The 3D calibration has a potential to eliminate the impacts of leaf angles on spectra and NDVI values and improve plant phenotyping results.

## Figures and Tables

**Table 1:** Specifications of the indoor desktop imaging system for image acquisition.

Camera Types	Parameters	Corresponding Settings
Hyperspectral camera	Camera model	acA 780–75 gm
	Spectrograph	Specim^®^ V10H
	Camera sensor type	Progressive scan CCD
	Camera gain mode	12-bit
	Camera focal length (mm)	Default lens focal length
	Camera spectral range (nm)	362–1043
	Camera field of view (°)	22.1
	Image spectral dimension (bands)	582
	Camera numerical aperture	F/1.4
	Image spatial resolution (pixels)	782
	Image integration time (ms)	16
	Scanning frame rate (fps)	60
	Scanning speed (mm/s)	6.35
RealSense camera	Depth module	Intel^®^ RealSense™ Depth module D435
	Baseline (mm)	50
	Left/right imagers type	Wide
	Depth FOV HD (°)	H:87 ± 3/V:58 ± 1/D:95 ± 3
	Depth FOV VGA (°)	H:75 ± 3/V:62 ± 1/D:89 ± 3
	IR projector	Wide
	IR projector FOV (°)	H:90/V:63/D:99
	Color sensor	OV2740
	Color camera FOV (°)	H:69 ± 1/V:42 ± 1/D:77 ± 1
	Image dimensions	720 × 1280 × 3

H-Horizontal FOV, V-Vertical FOV, D-Diagonal FOV.

**Table 2:** ANOVA results of soybean NDVI at different tilt angles and orientations.

Source	SS	df	MS	F	Prob > F
Columns	0.8195	8	0.1024	82.8310	0
Rows	0.0394	7	0.0056	4.5568	6.006 × 10^−5^
Interaction	0.0535	56	9.5507 × 10^−4^	0.7722	0.8846
Error	0.6233	504	0.0012		
Total	1.5358	575			

**Table 3:** ANOVA results of corn NDVI at different tilt angles and orientations.

Source	SS	df	MS	F	Prob > F
Columns	1.1583	8	0.1448	74.1286	0
Rows	0.5349	23	0.0764	39.1212	0
Interaction	0.1927	184	0.0034	1.7621	5.0877 × 10^−4^
Error	3.2345	1656	0.0020		
Total	5.1204	1727			

**Table 4:** The comparison of the original and estimated coordinates of 13 corner points.

	Original Coordinates (Pixels)		Estimated Coordinates (Pixels)	
Points	Rows	Columns	Rows	Columns
1	435	122	434	121
2	435	393	437	394
3	437	667	436	666
4	1097	667	1100	668
5	1755	667	1753	666
6	1755	429	1757	430
7	1752	119	1751	119
8	1092	122	1094	122
9	962	312	961	311
10	964	484	963	483
11	1251	479	1250	478
12	1246	312	1245	311
13	1152	395	1152	397

## Data Availability

The data presented in this research are available on request.
